# Characterization of aberrant pathways across human cancers

**DOI:** 10.1186/1752-0509-7-S1-S1

**Published:** 2013-08-12

**Authors:** Antti Ylipää, Olli Yli-Harja, Wei Zhang, Matti Nykter

**Affiliations:** 1 Department of Signal Processing, Tampere University of Technology, Korkeakoulunkatu 1, 33101 Tampere, Finland; 2 The University of Texas MD Anderson Cancer Center, 1515 Holcombe Blvd, Houston, TX 77030, USA; 3 Institute for Biomedical Technology, University of Tampere, Biokatu 8, 33520 Tampere, Finland

## Abstract

**Background:**

Cancer is a broad group of genetic diseases which account for millions of deaths worldwide each year. Cancers are classified by various clinical, pathological and molecular methods, but even within a well-characterized disease, there is a significant inter-patient variability in survival, response to treatment, and other parameters. Especially in molecular level, tumours of the same category can appear significantly dissimilar due to complex combinations of genetic aberrations leading to a similar malignancy. We extended the current classification methods by studying tumour heterogeneity at pathway level.

**Methods:**

We computed the rate of alterations in 1994 pathways and 2210 tumours consisting of eight different cancers. Using gene set enrichment analysis, each sample was computed a pathway aberration profile that reflected its molecular state. The profiles were analysed together to infer the characteristic aberration rates for each pathway within each cancer. Subgroups of tumours defined by similar pathway aberrations were identified using clustering analyses. The pathway aberration and gene expression profiles of the subgroups were consecutively compared across all eight cancer types to search for similar tumours crossing the standard classification.

**Results:**

We identified pathways and processes that were common to all cancers as well as traits that are unique to a cancer type or closely related cancers. Studying the gene expression patterns within the pathway context suggested potential alteration mechanisms. Clustering analysis revealed five clinically relevant subgroups of tumours in four cancers that exhibited significant differences in survival compared to others. The cross-cancer analysis of the subgroups resulted in the identification of tumours that shared potentially significant alterations.

**Conclusions:**

This study represents the first effort to extend the molecular characterizations towards pathway level descriptions across the family of cancers. In addition to providing a proof-of-concept for single sample pathway aberration analysis in this context, we present a comprehensive pathway aberration dataset that can be used to study pathway aberration patterns within or across cancers. Significant similarities between subgroups of different cancers on pathway and gene expression levels provide interesting hypotheses for understanding variable drug response, or transferring treatments across diseases by identifying common druggable pathways or genes, for example.

## Background

The development of cancer is an evolutionary process that is driven by the acquisition of somatic genetic mutations which give cells a selective advantage against non-mutated cells [1]. In order to become malignant cancer cells, normal cells need to acquire a set of mutations which confer "hallmark" traits, such as increased proliferation, immortality, and invasiveness [2]. Usually, a single mutation is not enough to result in malignant growth, but there are plenty of different combinations of mutations which can alter the expression biochemical pathways leading to the same phenotypic effect [3]. Acquiring these traits can be better described and understood as alterations in the balance of interaction networks of genes, proteins and other molecules, or pathways. Cancers can also be divided into clinically meaningful subtypes based on their gene expression patterns that may be indicative of response to a treatment, like Her2 positive breast cancers [4] or KIT positive gastro-intestinal tumours [5], for example. Many cancers have characteristic sets of somatic mutations that can be used to identify and classify the tumours [6]; few studies have even compared these across cancer types [7]. However, cancers are not commonly classified or studied based on the acquired traits or alterations to the pathways because of the added complexity. Instead, pathway level changes are often concluded for the tumour subgroups that have first been identified by other means. Currently, most established cancer types and cancer grading systems (such as Gleason score for prostate cancers [8] and WHO grades for tumours of the central nervous system [9]) are not even based on genetic markers but instead on clinical parameters and phenotypic observations.

Multi-institutional projects, such as The Cancer Genome Project (TCGA) and International Cancer Genome Consortium (ICGC), are already improving the cancer classification by systematically gathering and analysing unprecedented amounts of microarray and deep sequencing data from multiple cancers. These data have been used to identify clinically meaningful subtypes based on genomic and transcriptomic [9-16] or epigenomic profiles [17,18]. The standard approach of clustering samples based on only one type of data has recently been extended towards integration of multiple data types [19,20]. Common practice is to follow up genomic and transcriptomic analyses by probing the frequently altered pathways in each identified subtype to infer the unique systems level characteristics of each subtype. Altered pathways can be identified by statistically combining knowledge on pathways' constituent genes, and their genomic (mutations, copy numbers) and/or transcriptomic (expression) state in the tumours [21]. Recent increase in availability of microarray and deep sequencing data has made it also possible to identify the extent and the frequency at which pathways are aberrant in different cancers and cancer subtypes. There is currently great interest in extending characterizations and subtyping into systems level. One of the main goals is to improve poor drug response rates by matching drugs with the specific pathway alterations of the patient's cancer subtype [22].

We hypothesize that classifying tumours based on clinical, phenotypic, or genomic markers may not be as informative as using pathway alterations since different tumours may appear similar and the molecular mechanisms to malignancy are undoubtedly variable. Furthermore, it is extremely difficult to predict the effect of DNA level changes (*e.g*. mutations, copy number changes, methylation levels) to the phenotype, and therefore we analysed data in the context that is as close to the phenotype as possible, the pathways. In this paper, we analyse multiple TCGA expression datasets from systems perspective. Using pathway data from five databases (1994 pathways) and expression data from eight cancers (2210 samples), we first infer the aberrant pathways in individual tumours, thus defining their pathway aberration profiles. Based on these profiles, we define a comprehensive catalogue of pathway alterations and their frequencies in respective cancers. By clustering the pathway aberration profiles, we are able to uncover clinically meaningful subtypes of cancers that have not been reported from TCGA cancer types by earlier studies. Finally, we compare the subgroups of different cancers together to find unexpected similarities on both pathway and gene expression level.

## Methods

### Gene expression data analysis and quality control

**Figure 1:**
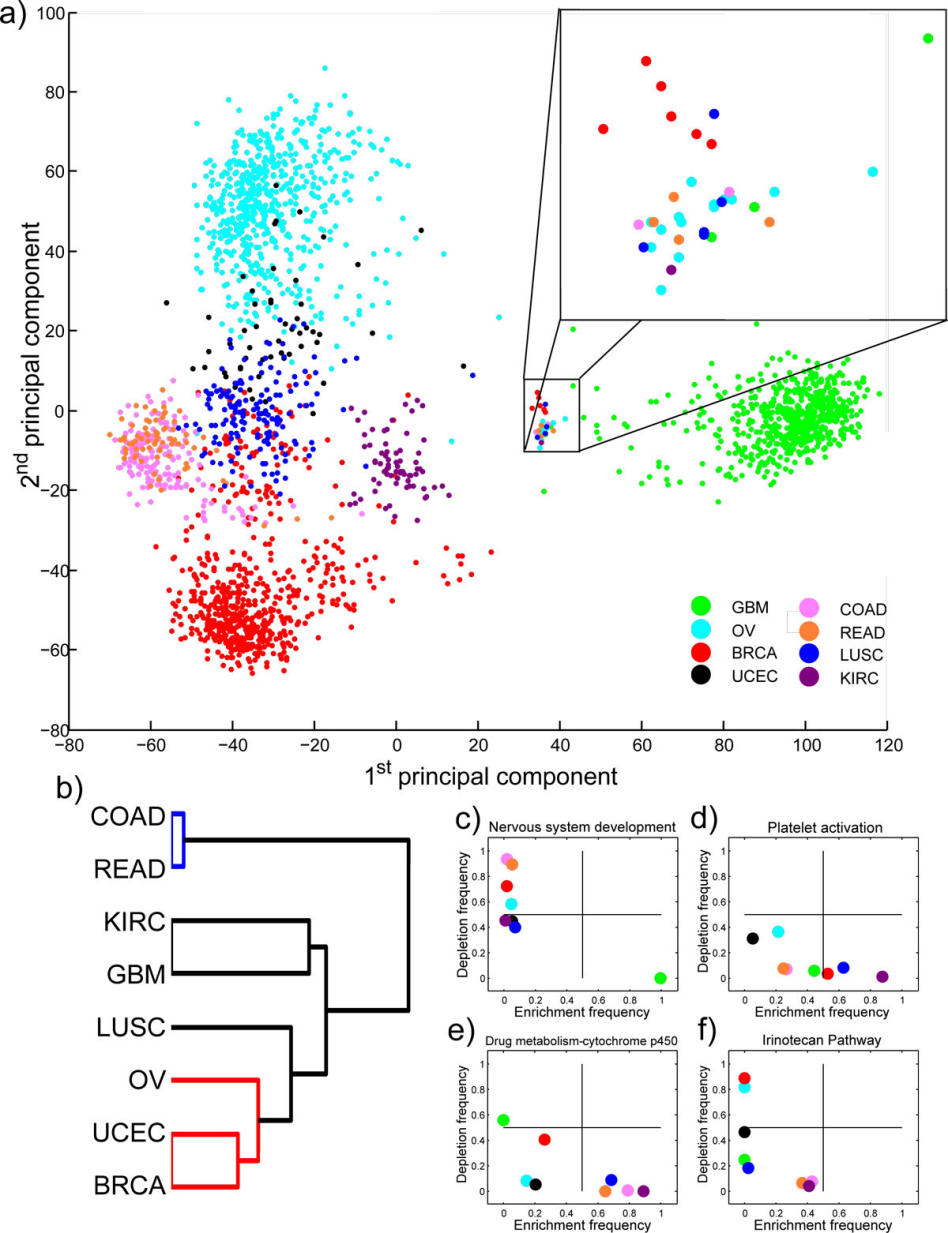
**Similarities and differences of tumour types in gene expression and pathway levels**. a) Two first principal components of the gene expression data from all the tumours are plotted. Colours denote the eight different cancer types. Blow-up panel highlights a distinct group of samples from almost all cancer types b) Hierarchical clustering of the pathway aberration profiles indicates that colon and rectal cancers (blue branch) are alike. Gynaecological cancers (red branch) are also similar on pathway level c) Nervous system development is enriched in nearly all GBM, but rarely in any other cancer type. There is a considerable variation in enrichment and depletion frequency in d) Platelet activation pathway e) Drug metabolism through cytochrome p450 and f) Irinotecan pathway.

Using TCGA data portal, we downloaded (on 3/8/2012) all of the dual-channel mRNA expression array data (Agilent 244K TCGA Custom 1-3) (n = 2365) from the eight cancer types that have been characterized by TCGA project (see Table 1). We used "level 3" data which corresponds to pre-processed and interpreted expression signals for each gene. These data had been normalized against Stratagene Universal Reference RNA and Lowess normalization had been applied on a per-gene basis by the TCGA. Log ratios of gene expressions were finally obtained for 17,814 genes. Expression values were quantile normalized for cross-sample variation for principal component analysis (PCA). After checking for consistency of gene expression profiles PCA analysis, we removed all duplicate samples so that each sample represents a unique case. We also removed data from patient IDs 'TCGA-07-0249' and 'TCGA-AV-A03E' that were found in many duplicates, had a distinct gene expression signature, had no meta data, and most alarmingly were annotated to many different cancers (see blow-up in Figure 1a). Removing the data from 155 duplicate or bad samples, we arrived at gene expression profiles of 2210 unique samples.

**Table 1:** Expression data

Symbol	Cancer type	Number of samples
GBM	Glioblastoma multiforme	582

OV	Ovarian cancer	582

BRCA	Breast cancer	534

COAD	Colon adenocarcinoma	162

LUSC	Lung squamous cell carcinoma	154

UCEC	Uterine corpus endometrial adenocarcinoma	54

READ	Rectal adenocarcinoma	70

KIRC	Kidney clear cell renal carcinoma	72

### Acquisition of the pathway information

We acquired gene set data from five databases (Biocarta (http://www.biocarta.com/genes/index.asp, 4/19/2011), WikiPathways (http://wikipathways.org, 1/3/2011), Pathway Commons (http://www.pathwaycommons.org, 11/30/2010), Gene Ontology Biological Processes v3.0 (http://www.geneontology.org, 1/3/2011), and Kyoto Encyclopedia of Genes and Genomes (http://www.genome.jp/kegg/pathway.html, 6/14/2010)), totalling over 2500 unique signalling or metabolic pathways or genes annotated to the same biological process. The gene sets were combined into a mutually compatible form by re-annotating the gene identifiers to a common namespace (HUGO nomenclature). Topologies of the pathways were not considered in any way. Gene sets with less than 10 or more than 1000 constituent genes were filtered out resulting in 1994 sets of interrelated genes that we call pathways hereafter (Table 2).

**Table 2:** Pathway data

Symbol	Database	Number of pathways
KEGG	Kyoto Encyclopedia of Genes and Genomes	186

BIOCARTA	Biocarta	214

PWC	Pathway Commons	656

GO	GeneOntology Biological Processes	808

WIKIPW	WikiPathways	130

### Computing pathway aberration profiles

To arrive with pathway aberration scores corresponding to enrichment and depletion of each pathway, we computed gene set enrichment scores inspired by the GSEA method by Subramanian *et al. *[21] for each pathway in each sample individually. These scores reflect the degree to which a pathway's genes are up- or downregulated compared to a reference in a sample. First we rank ordered the list of all measured genes based on their normalized expression difference against a reference (as described above). Then, walking-down the list of genes, we label each gene as 1 if it belongs to the pathway or 0 if it does not belong to the pathway. Starting from the most upregulated gene results in a score for enrichment and starting from the most downregulated gene a score for depletion. Drawing analogy to analysis of Receiver Operating Characteristic (ROC) curves, we derive the fraction of 1's vs. the fraction of 0's at each position of the list and compute the Area Under Curve (AUC) statistic which describe how far up or down the list are the pathway's genes. To estimate the statistical significance of the AUC, we permute the list of ordered genes and recompute the statistic for the permuted data 1000 times to generate a null distribution for the AUC. This method for generating null distributions was chosen, because in the single sample analysis, more complex models are difficult to justify as there is no meaningful way to evaluate correlations with phenotypes. Empirical confidence scores of the observed AUC are then calculated relative to the null distribution. By iterating the algorithm for each sample and each pathway individually, we obtained two scores for each sample-pathway combination, corresponding enrichment and depletion. Smaller value of the score indicated more significant trend. In order to avoid being overly conservative for an exploratory method, we chose not to control for the amount of false positives due to multiple testing problem. Combining the enrichment and depletion scores for each pathway in each sample, we created pathway aberration profiles that describe all the pathway aberrations in each sample. This was done by taking the smaller of enrichment and depletion scores, and transforming it into log_2 _space if it was enrichment and -log_2 _space if it was depletion.

### Clustering of pathway aberration profiles

To investigate the similarities and differences between cancers on pathway level we hierarchically clustered the means of pathway aberration profiles with Euclidean distance metric and Ward's linkage method. To identify homogeneous subtypes, a two way clustering of the aberration profiles across samples and pathways was done using hierarchical clustering with the same distance metric and linkage method. Distinct branches of 20-30 samples were identified from the dendrogram and further studied as subgroups. Clustering of the identified subgroups was done using the same methodology and features consisting of mean enrichment and depletion frequencies within each subgroup.

### Statistical analyses

Associations between patient survival and subtype were computed with Mantel-Cox test of difference of Kaplan-Meier survival estimators. Associations between the subgroups and previous molecular subtype characterizations were computed using Fisher's exact test. Differential gene expressions were computed using Wilcoxon rank sum test. A *p *-value of 0.05 was considered the threshold of statistical significance in all tests. All analyses were made with Matlab version R2010b (MathWorks, Natick, MA).

## Results and discussion

### Identification of common and disease-specific sets of pathway aberrations in eight cancers

The 2210 gene expression profiles consisted of eight different cancer types (see Table 1) provided by the TCGA. Three cancers are represented by over 500 samples each, two cancers by at least 150 samples, and three cancers are represented by at least 50 samples each. Principal component analysis indicated that the gene expression profiles of colon and rectal carcinomas are very much alike, similarly as ovarian and uterine tumours, whereas glioblastoma has the most distinct gene expression pattern (Figure 1a). A very small subset from all cancers, except endometrial, closely resembled each other in gene expression level (blow-up panel in Figure 1a). Closer inspection revealed that these were data from only two patients according to patient ID numbers. The data from these patients were removed as well as all duplicate samples so that each sample represented a unique case.

Using a collection of 1994 pathways from five different databases (see Table 2), we investigated the similarities and differences between cancers in pathway level. Instead of pooling the data from each cancer type first, we computed the enriched and depleted pathways for each sample individually (Additional File 1). We then combined the pathway enrichment and depletion scores into pathway aberration profiles for each cancer type (see Methods), and hierarchically clustered them (Figure 1b). On pathway level, colon and rectal carcinomas have very few differences (blue branch). Also, the gynaecological cancers (BRCA, UCEC, and OV) clustered closely together (red branch), as could be expected. By comparing the average aberration rates across all cancer types, we observed that many of the biological processes considered as cancer hallmarks [2] are frequently aberrant in all tumour types. For example, GO terms Inflammatory response (81%), Immune response (80%), Cell-to-cell signalling (77%), Cell-to-cell adhesion (76%), and Cellular homeostasis (67%) were among the most frequently enriched processes, whereas DNA replication (98%), Regulation of cell cycle (98%), were among the most frequently depleted (Additional File 2).

However, by ranking the pathways according to their variability in alteration frequencies between cancers, we identified several pathways that were altered very frequently within one or few cancer types and only rarely in other cancers. These pathways can give rise to the observed differences in physiological and phenotypic properties across cancers, or they may only reflect the differences between host tissues or cells of origin, especially since there were no tissue-specific normal references available. For example, GO term Nervous system development which is enriched in 99.5% of GBM's, but hardly ever in other tumour types, is likely a cell type specific pathway rather than malignant alteration (Figure 1c). However, we identified processes that we think are actually more related to cancer than normal cell physiology. For example, platelet activation is enriched in 87% of kidney carcinomas, but only in 6% of uterine adenocarcinomas, which may translate to differences in tumour haemostatic activity or formation of cancer metastases through emergence of platelet-tumour cell aggregates [23] (Figure 1d). Another potentially interesting observation is the major difference in enrichment of the drug metabolism by Cytochrome p450 pathway which is closely related to multiple drug resistance, and also represents a potential therapeutic target [24]. It is highly enriched in cancers of the kidney, lung, colon and rectum (64%-89%), lowly enriched in gynaecologic cancers (ovarian, breast, and endometrial) (15%-26%) and never in glioblastoma. In contrast, the pathway is actually depleted in 56% of GBMs (Figure 1e). There is also a significant difference in the enrichment and depletion frequencies of Irinotecan pathway which describes the biotransformation of the chemotherapy prodrug irinotecan to form the active metabolite which inhibits DNA topoisomerase I [25]. The drug is used in the treatment of many different cancers, but there is large interpatient variability in response to it. The pathway is very frequently depleted in ovarian and breast cancers (81% and 89%, respectively, but rarely in cancers of kidney, colon, and rectum (4%, 8%, and 7%, respectively) (Figure 1f).

### Pathway aberration profiles identify clinically significant subtypes in glioblastoma, breast cancer, colon cancer, and ovarian cancer

Common molecular subtypes have already been identified in many of the cancers before [11,13,14,17]. To find out how these related to pathway aberrations, and to identify new subgroups based on pathway level changes, we hierarchically clustered the samples into small and homogeneous clusters characterized by a unique set of pathway aberrations (Additional File 3, Figure 2, **Figures S1-S7 in **Additional File 4,). We divided the eight tumour types into 62 subgroups each consisting of 1.5-65% of the samples. Subgroups were also compared to the previously established molecular subgroups or pathological grading and staging systems where available. In general, our subgroups reflected the molecular classifications, but not the tumour grades and stages. Clinical relevance of the subgroups was investigated by comparing the survival estimators; however, the combination of modest number of samples in some cancers (especially KIRC, READ, and UCEC) and very short follow-up times for many patients hindered the strength of this analysis. Associations to other potentially relevant clinical variables were omitted due to poor quality or complete lack of available metadata.

**Figure 2:**
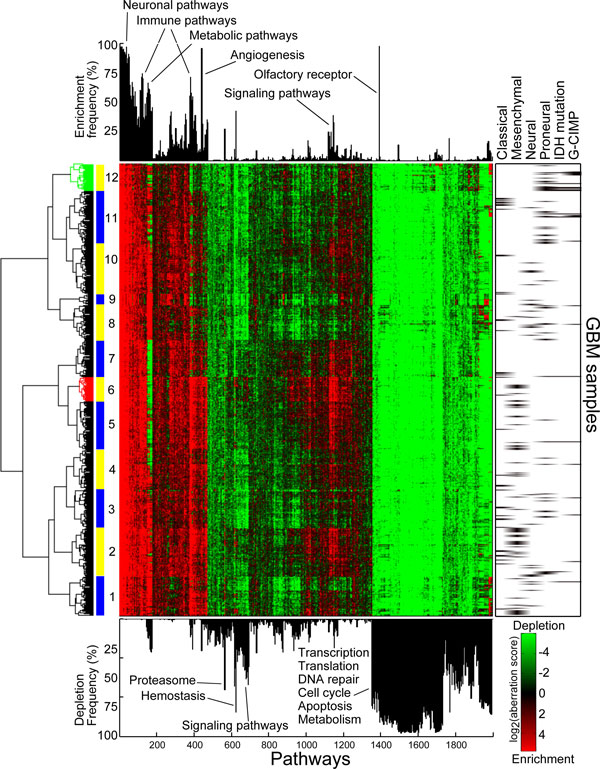
**Clustering of glioblastoma pathway aberration profile**. Pathway aberration matrix of GBM shows enriched (in red) and depleted pathways (in green). The bar plot on top of the matrix shows the frequency of enrichment for each pathway, and the bar plot below shows the frequencies of depletion. Some of the most frequently aberrant pathways and processes are annotated to the figure. Hierarchical clustering of the matrix resulted in 12 subgroups (marked in alternating blue and yellow bars, first subgroup being marked with the first blue bar in the bottom), two of which were clinically relevant (subgroup 6 in red and subgroup 12 in green). Some of the subgroups corresponded to the pre-existing molecular classifications in right.

**Figure 3:**
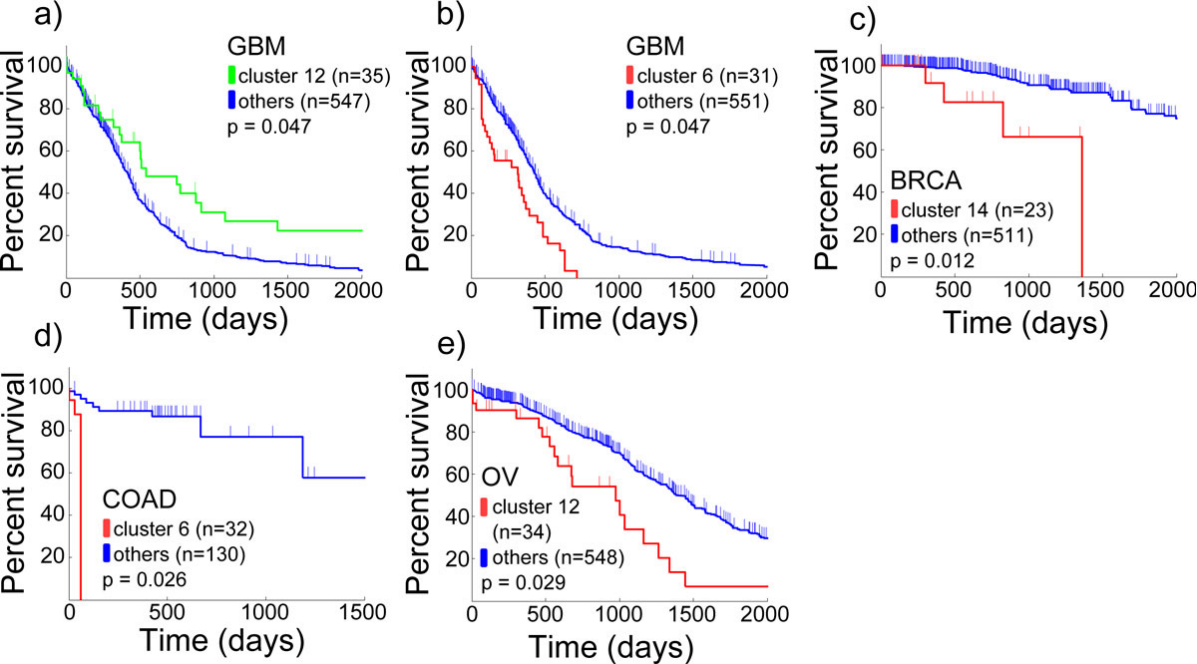
**Comparison of Kaplan-Meier survival estimators for clinically significant subgroups of patients**. Kaplan-Meier survival estimators for subgroups that have a significant survival difference compared to the rest of the cohort a) patients with tumours in GBM cluster 12 (green) have a significantly increased survival estimate compared to other GBM samples (blue) (p = 0.047) b) patients in GBM cluster 6 (red) have a significantly worse survival estimate compared to others (blue) (p = 0.047) c) BRCA cluster 14 (red) contains patients with worse survival (p = 0.012) d) COAD cluster 6 (red) contains patients with worse survival (p = 0.026) e) OV cluster 12 (red) contains patients with worse survival (p = 0.029)

**Figure 4:**
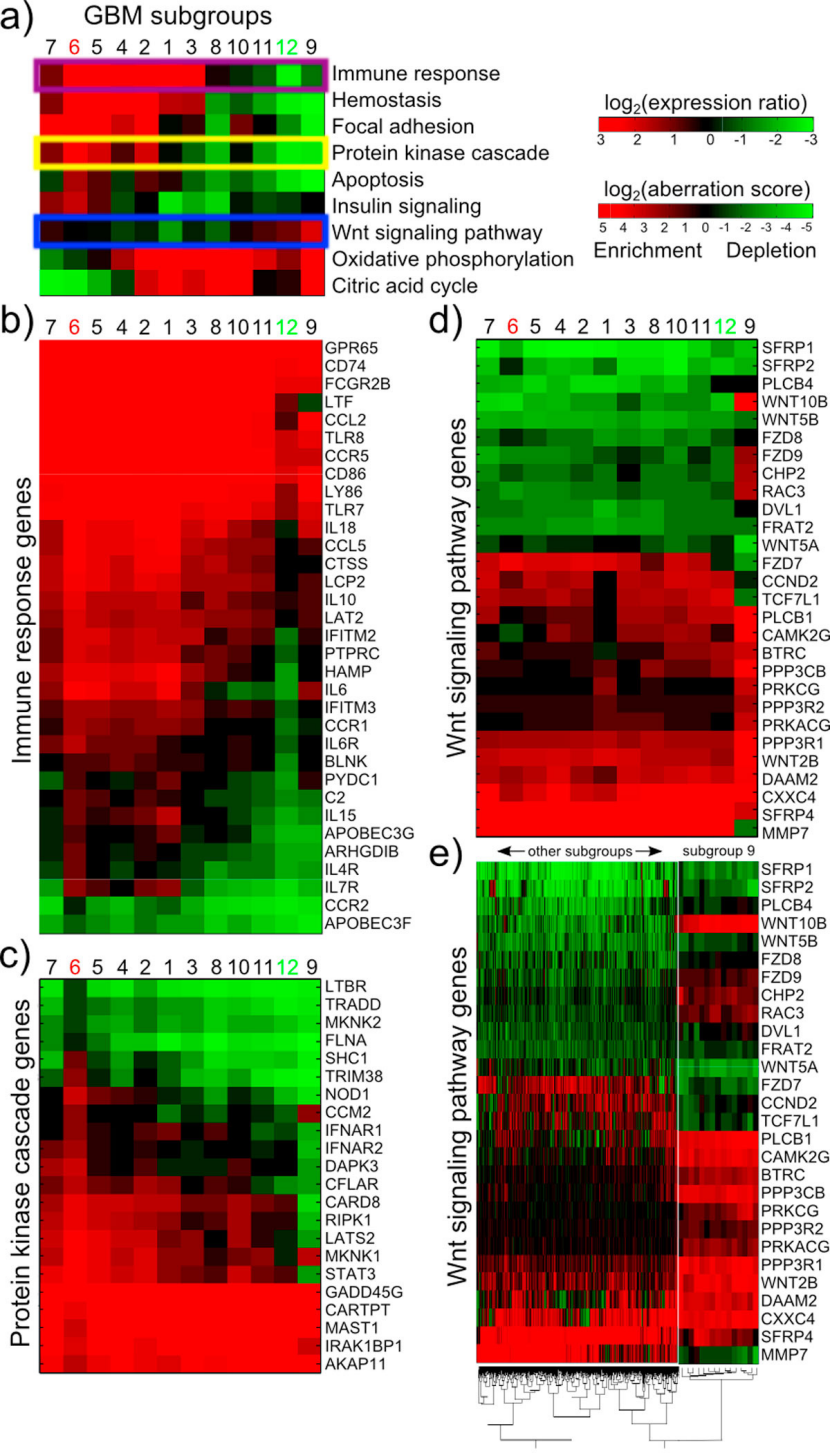
**Differential pathway and gene expression patterns in glioblastoma subgroups**. a) Alternating pathway aberration patterns for nine pathways are shown for the GBM subgroups. Highlighted are Immune response pathway (purple) that is depleted in subgroup 12, Protein kinase pathway (yellow) enriched in subgroup 6, and Wnt signalling pathway (blue) enriched in subgroup 9. Shown in panels below are mean expression levels in subgroups for the genes in three highlighted pathways: b) Immune response, c) Protein kinase cascade and d) Wnt signalling pathway. E) Expression levels of Wnt signalling pathway genes in individual samples demonstrate the consistency of gene expression patterns within an enriched subgroup.

**Figure 5:**
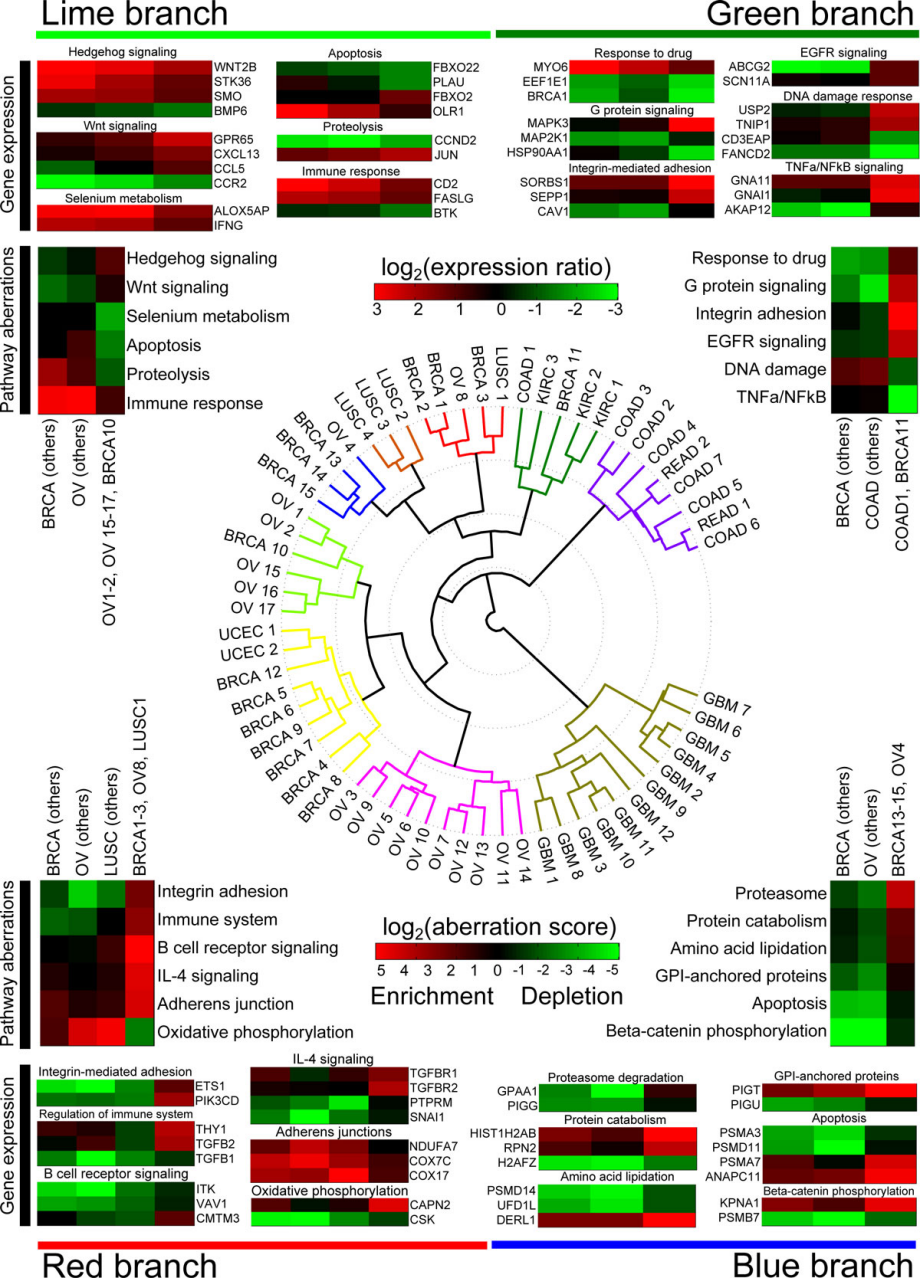
**Pathway and gene expression level comparison of subgroups across all cancers**. Polar dendrogram in the centre shows how subgroups of different cancers hierarchically cluster together. GBM subgroups (olive branch) all cluster together, but there are also mixed clusters, such as the lime, green, red and blue branches, containing subgroups of few different cancers. Some of the key differences between subgroups in mixed branches are highlighted for these four branches. For example, TNFa/NFkB signalling is enriched in the green branch (upper right corner) subgroups COAD1 and BRCA11 in contrast to other BRCA and COAD tumours. Few of the most differentially expressed genes in this pathway between these subgroups and other tumours of the same type were GNA11, GNAI1, and AKAP12 shown in upper right corner.

Interestingly, based on the clustering of GBM pathway aberration profile (Figure 2), we found one subgroup that was significantly less lethal (*p *<0.05) (Figure 3a) than the others, and one that was more lethal (*p *<0.05) (Figure 3b). Tumours in the less lethal subgroup were enriched of Proneural subtype [11] (*p *<4.5e-5), and concordantly with IDH mutations (*p *<5.1e-5) and glioma-CpG Island Methylator Phenotype (G-CIMP) [17] (*p *<1.7-e6). Since TCGA's GBM cohort currently is considerably larger compared to the one used in the previous studies, not all of tumours were annotated to these groups. Our results encourage investigating the remainder of the tumours in this subgroup for the associated features of Proneural and G-CIMP tumours. The most common molecular subtype in the more lethal subgroup was Mesenchymal [11] (*p *<0.02). Additionally, from BRCA pathway aberration profile (Figure S1 in Additional File 4), we discovered a more aggressive subgroup (*p *<0.05) (Figure 3c) that was enriched in Her2 positive tumours [14] (*p *<1.4e-5). Some of the other subgroups were also enriched in tumours annotated to specific molecular subgroups (Basal-like, LuminalA and LuminalB) underlining the differences of these subtypes not only on mutation and gene expression level but also functionally. For example, subgroups 3 and 4 that consisted of a significant portion of the Basal-like tumours (*p *<1.8e-9 and *p *<2.4e-7, respectively) were not more aggressive than others, in agreement with previous findings of Basal-like tumours [14]. Based on pathway aberration profiles of colon (FigureS2inAdditional File 4) and ovarian (Figure S3 in Additional File 4) cancers, we identified two additional aggressive (*p *<0.05) subgroups (Figure 3d-e). Neither group were associated to the tumour stages. No subgroups with significantly different survival estimators were found in READ (FigureS4 inAdditional File 4), LUSC (FigureS5 inAdditional File 4), KIRC (FigureS6 inAdditional File 4), and UCEC (FigureS7 inAdditional File 4), probably also due to the smaller sample sizes and shorter patient follow-up periods.

Investigating the pathway aberration differences between the subgroups, and the molecular mechanisms that cause these aberrations may offer interesting insight into the diseases. In Figure 4a, we show the mean pathway aberration profiles for nine pathways that are differentially aberrant in the 12 GBM subgroups. This analysis revealed that the tumours in the less lethal GBM subgroup 12 (Proneural/G-CIMP enriched) differed significantly on a number of pathway aberration frequencies. For example, GO category Immune response was enriched in 0% and depleted in 97% of the less lethal tumours, whereas the aberration frequencies in other tumours were 54% and 9% for enrichment and depletion, respectively. Apoptotic and haemostatic pathways were also significantly depleted compared to others. The tumours in the more lethal GBM subgroup 6 differed from the others the most radically in aberration rate of signalling cascades, and metabolic pathways. To understand the molecular mechanisms behind these pathway level changes, we investigated the mean gene expression levels in three highlighted pathways: Immune response depleted in subgroup 12 (Figure 4b), Protein kinase cascade enriched in subgroup 6 (Figure 4c), and Wnt signalling pathway enriched in subgroup 9 (Figure 4d). The genes that cause the differential pathway aberration rates in these subgroups are clearly observed: Immune response genes IFITM2-3, PTPRC, HAMP, CCR1, IL6R, and BLNK, for example, are exclusively underexpressed in subgroup 12 causing the depletion. Several protein kinase cascade genes are not underexpressed in subgroup 6, in addition to exclusive overexpression of SHC1, TRIM38, NOD1, and others, causing the enrichment. In Wnt signalling pathway, enriched only in subgroup 9, we observed especially WNT10B, FZD9, CHP2, and RAC3 overexpression and FZD7, TCF7L1, and MMP7 underexpression, whereas in other subgroups their expression changes were the opposite. To further investigate the consistency of the differential gene expression levels within subgroups, we clustered the expression ratios of the genes in Wnt signalling pathway in GBM (Figure 4e). Samples in subgroup 9 formed a separate cluster from the other samples, and from the individual gene expression pattern we observed that the most prominent genes listed above, such as WNT10B, were indeed very consistently differentially expressed in this subgroup compared to others.

In Figure S8ainAdditional File 4, we show the mean pathway aberration profiles for five pathways that are differentially aberrant in the three KIRC subgroups. The expression levels of the glycolysis-related genes indicate how exactly this pathway is depleted in KIRC subgroup 2 and enriched in subgroup 3. DPYS, UPB1 and PPAP2B are consistently upregulated in samples where glycolysis pathway is not enriched, whereas a group of eight downregulated genes (ACLY, PFKFB3, MLXIPL, SLC35D1, TPI1, RPIA, AMPD2, and PFKB4) are a characteristic of the glycolysis-enriched subgroup 3 in addition to a fair amount of upregulated genes, including subunits of NADH dehydrogenase complex, ATP synthase complex, cytochrome c oxidase, and ubiquinol-cytochrome reductase. In FigureS8b inAdditional File 4, tumours in the more lethal OV subgroup 12 were particularly enriched in metabolic, immune response, transcriptional and translational pathways. Metabolic and immune pathways were also enriched in the more lethal BRCA subgroup 14 (in FigureS8c inAdditional File 4). BRCA subgroups 3 and 4 that consisted of a significant portion of the Basal-like tumours were highly enriched in adaptive immune system processes (Adaptive immune response GO category enriched in 92% of the tumours in this subgroup compared to 20% in other tumours) such as Lymphocyte activation, and TNFa/NFkB signalling [26], which may have significant clinical implications. Other pathway-level features of the subgroup included less frequently enriched secretion, and more frequently depleted catabolic pathways. The more lethal COAD subgroup 6 was enriched of transcriptional and metabolic pathways (in FigureS8d inAdditional File 4). Collectively, some of the most variably aberrant pathways in these cancers included metabolic pathways such as oxidative phosphorylation, transcriptional and translational pathways, immune system related pathways, processes such as haemostasis, apoptosis and cell proliferation, and signalling pathways such as TNFa/NFkB signalling (Figure S8a-g inAdditional File 4). This may indicate that not all of these processes are necessary to the cancer cells, or that there exist alternative molecular mechanisms to acquiring the phenotypes that are described by these pathways.

### A family tree of cancer: Pathway level comparison reveals functionally similar subgroups across cancer types

Fuelled by discovery of similar pathway level characteristics between subgroups of different cancers, and previous results indicating that there are relevant similarities between cancer subtypes, such as those between Basal-like breast cancer and high-grade serous ovarian cancer [14], we further clustered all subgroups together based on the pathway aberration frequencies in each group (Figure 5). We wanted to find subgroups of different cancers that shared common pathway aberration profiles. The clustering indicated that all GBM subgroups (olive branch) are alike, and none of them share considerable amount of pathway aberrations with subgroups of any other cancer. This may also be due to enrichment and depletion of cell-type specific pathways. Most of the colon and rectal tumours were clustered together (purple branch) with the exception of COAD subgroup 1 that clustered with kidney cancers and BRCA subgroup 11. Other mixed type branches included the red, blue and lime branches which consisted of BRCA, OV, and LUSC subgroups. We did not include the three KIRC subgroups in further analysis of the green branch, because there were no other KIRC samples to compare with in other branches, neither did we not consider the yellow branch with two endometrial subgroups a mixed branch.

In the four mixed branches (lime, green, red, and blue), we compared the pathway aberration differences between the mixed tumours to the tumours of the same type in other branches. In the upper left corner of Figure 5, we show six pathways that are differently aberrant between the five OV subgroups and BRCA subgroup 10 in the lime branch comparing to the rest of OV and BRCA subgroups. Immune response and selenium metabolism were the most strikingly differently aberrant pathways. For the six pathways, we show the expression differences of few differently expressed genes (*p *<0.01) which may provide clues to understanding why the pathways are altered. For example in immune response pathway, CD2 and FASLG are not overexpressed in the lime branch subgroups compared to other BRCA and OV tumors. Similarly BTK is underexpressed in the lime branch subgroups.

In the green branch, COAD1 and BRCA11 share the depleted TNFa/NFkB signaling pathway, and enriched integrin adhesion pathway in comparison to other BRCA and COAD tumors. Significant differences in some of the genes in the selected pathways are observed, for example, in TNFa/NFkB pathway AKAP12 is underexpressed in BRCA and COAD tumors excluding COAD1/BRCA11 where GNAI1 and GNA11 are overexpressed. The red branch consisted of three types of cancers that share the enrichment in integrin adhesion, immune system, B cell receptor signaling, IL-4 signaling, and adherens junctions in contrast to the rest of the BRCA, OV, and LUSC tumors. Interestingly, TGFB1, TGFB2, TGFBR1, and TGFBR2 are all upregulated in these tumors. Subgroups of OV and BRCA in the blue branch are characterized by the frequently depleted apoptosis and beta-catenin phosphorylation pathways.

## Conclusions

Following huge efforts to measure the genomes and transcriptomes of different cancers, this study represents the first effort to extend the current molecular characterizations towards comparative and pathway level descriptions across the family of cancers. Studying large collections of tumour samples at pathway level enabled us to create a comprehensive catalogues of altered pathways from where we inferred the characteristic aberrations for each cancer. As such, this study is also the first proof-of-concept study for utilizing single sample pathway aberration analysis in this context. Importantly, our approach adds another layer of information on top of the classical markers retaining the option to study gene expression or other genomic features in the context of pathways as well. Based on the pathway aberration profiles alone, we identified clinically significant subtypes of glioblastoma, breast cancer, colon cancers, and ovarian cancer. In contrast to subtypes identified using genomic data, phenotypic characteristics of our subtypes can be easily hypothesized from their unique pathway aberrations. We also identified significant similarities between subgroups of different cancers on pathway and gene expression levels which provide interesting avenues for understanding variable drug response or transferring treatments across cancer types by identifying common druggable pathways or genes, for example. These results demonstrate the applicability of our approach, and the value of the aberration data as a resource for future investigations where integrating e.g. copy number, mutation, and epigenetic data to our results should provide plenty of intriguing insight.

## Competing interests

The authors declare that they have no competing interests.

## Authors' contributions

AY planned the study, carried out the bioinformatics analyses, and drafted the manuscript, WZ and OY-H participated in design and coordination, MN conceived the study and participated in its design and coordination and helped to draft the manuscript. All authors read and approved the final manuscript.

## Supplementary Material

Additional file 1 Pathway aberration scores for each sample Click here for file

Additional file 2 Pathway aberration frequencies for each cancer type Click here for file

Additional file 3 Subgroup classifications for each sample Click here for file

Additional file 4 Supplementary figures Click here for file

## Declarations

Publication of this article was supported by the following agencies and grants: Academy of Finland (project no. 132877, 251790), Emil Aaltonen Foundation, Finnish Funding Agency for Technology and Innovation Finland Distinguished Professor Programme, and National Cancer Institute grant no. U24CA143835.

This article has been published as part of BMC Systems Biology Volume 7 Supplement 1, 2013: Selected articles from the 10th International Workshop on Computational Systems Biology (WCSB) 2013: Systems Biology. The full contents of the supplement are available online at http://www.biomedcentral.com/bmcsystbiol/supplements/7/S1.
