# Supplementary figures and images for: Characterization of aberrant pathways across human cancers
Source: BMC Syst Biol. 2013 Aug 12;7(Suppl 1):S1. doi: 10.1186/1752-0509-7-S1-S1 (PMC3750561; doi:10.1186/1752-0509-7-S1-S1)

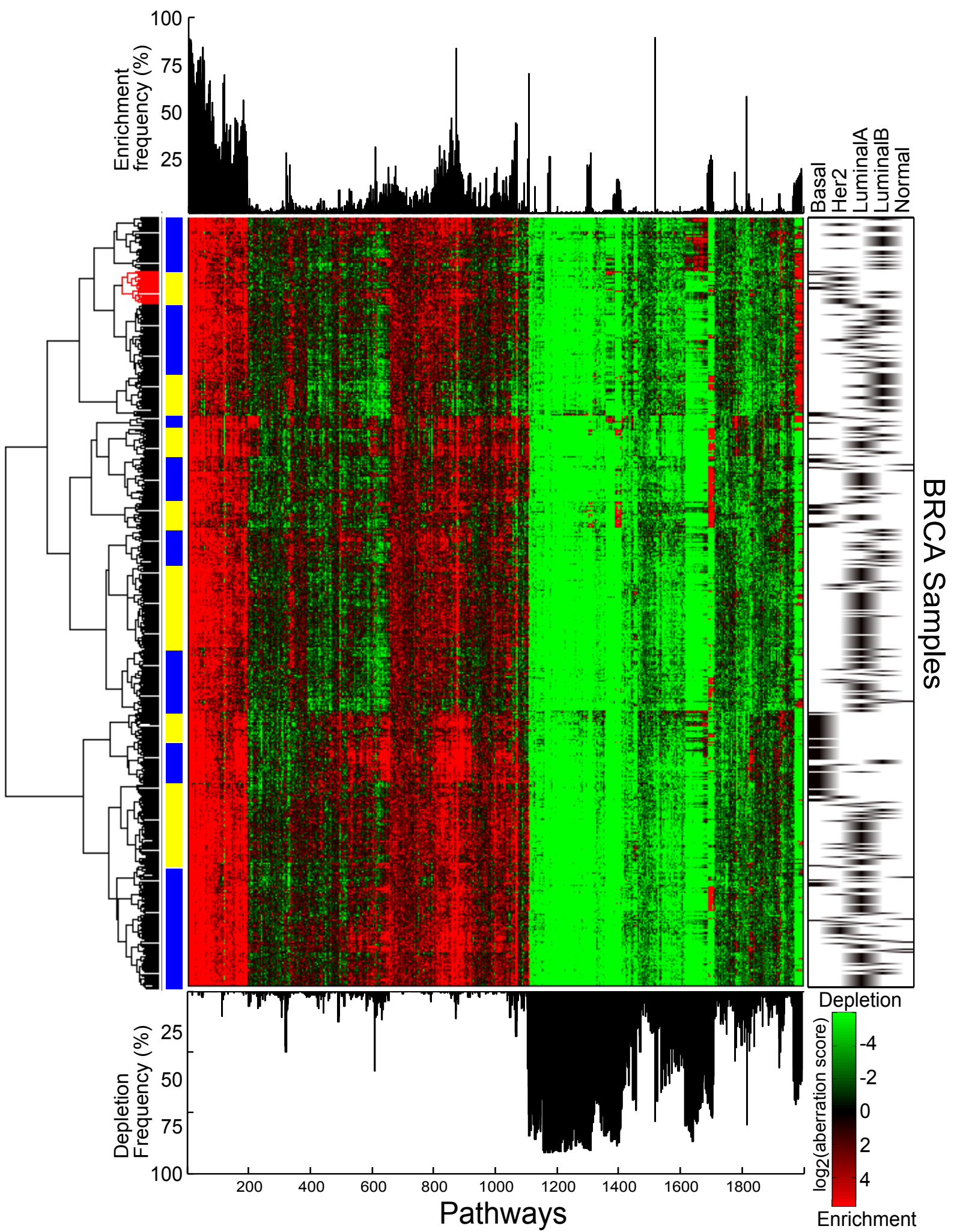

Supplement: Additional file 4 — Supplementary figures [file 1752-0509-7-S1-S1-S4.zip › figS1.pdf]

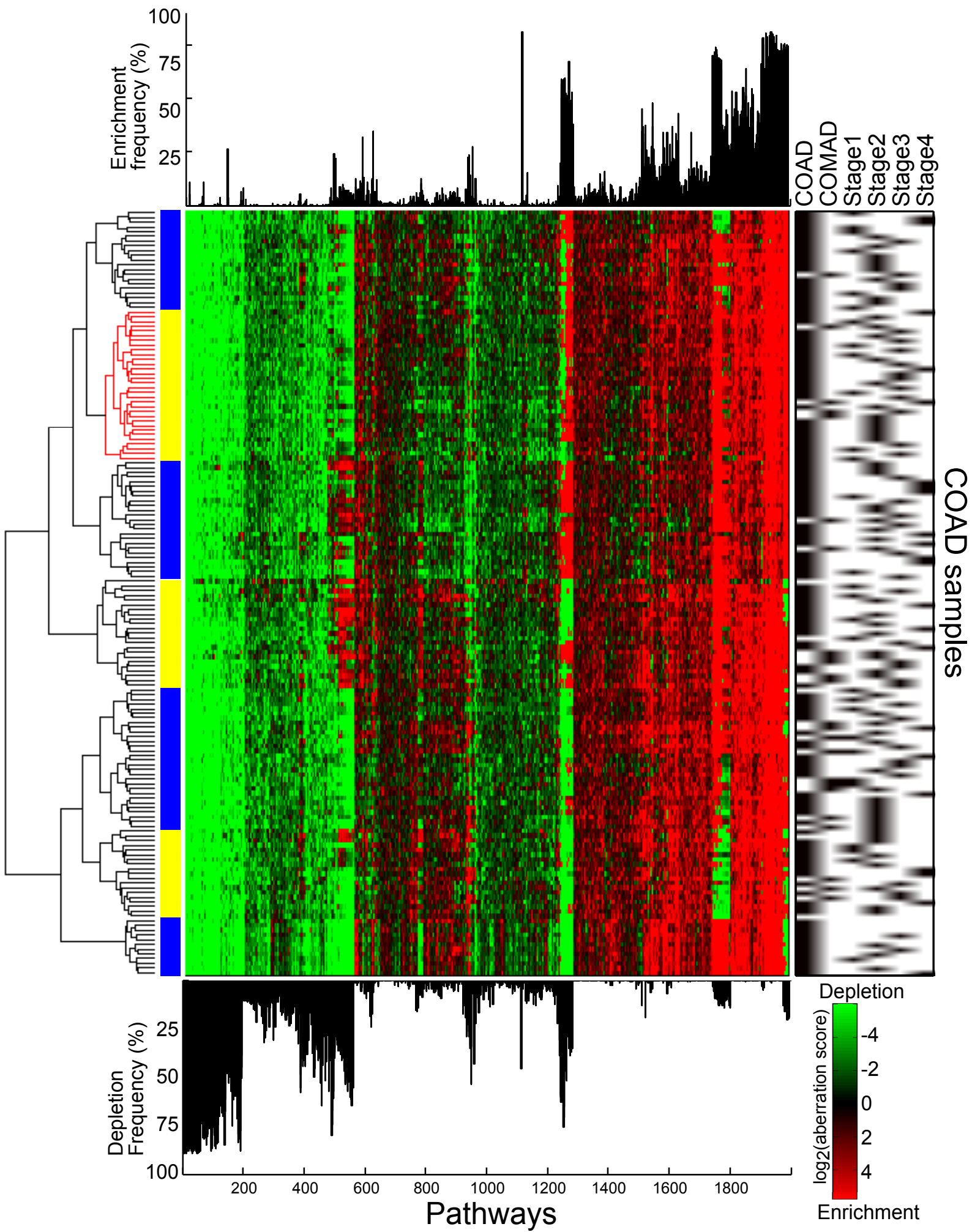

Supplement: Additional file 4 — Supplementary figures [file 1752-0509-7-S1-S1-S4.zip › figS2.pdf]

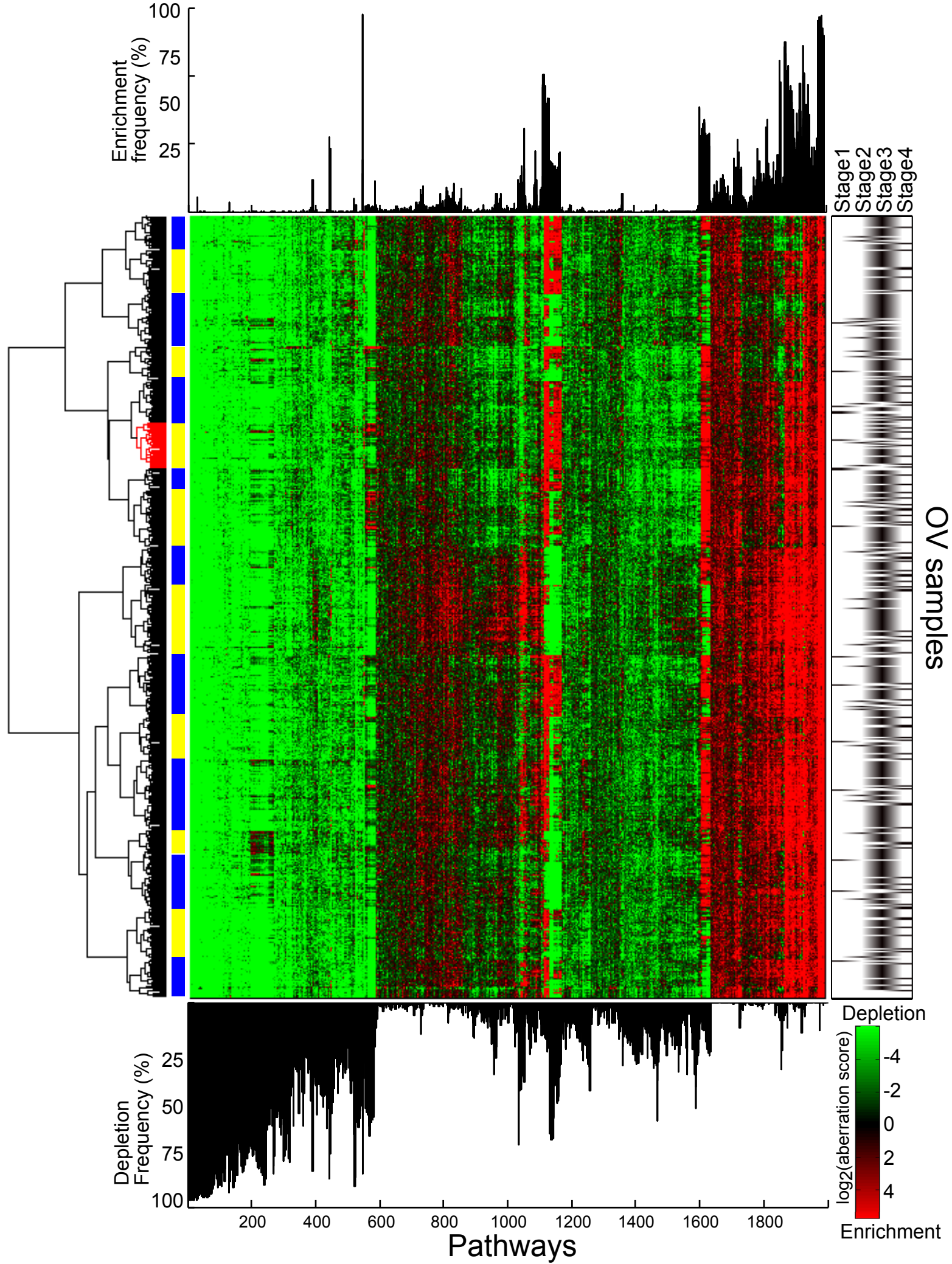

Supplement: Additional file 4 — Supplementary figures [file 1752-0509-7-S1-S1-S4.zip › figS3.pdf]

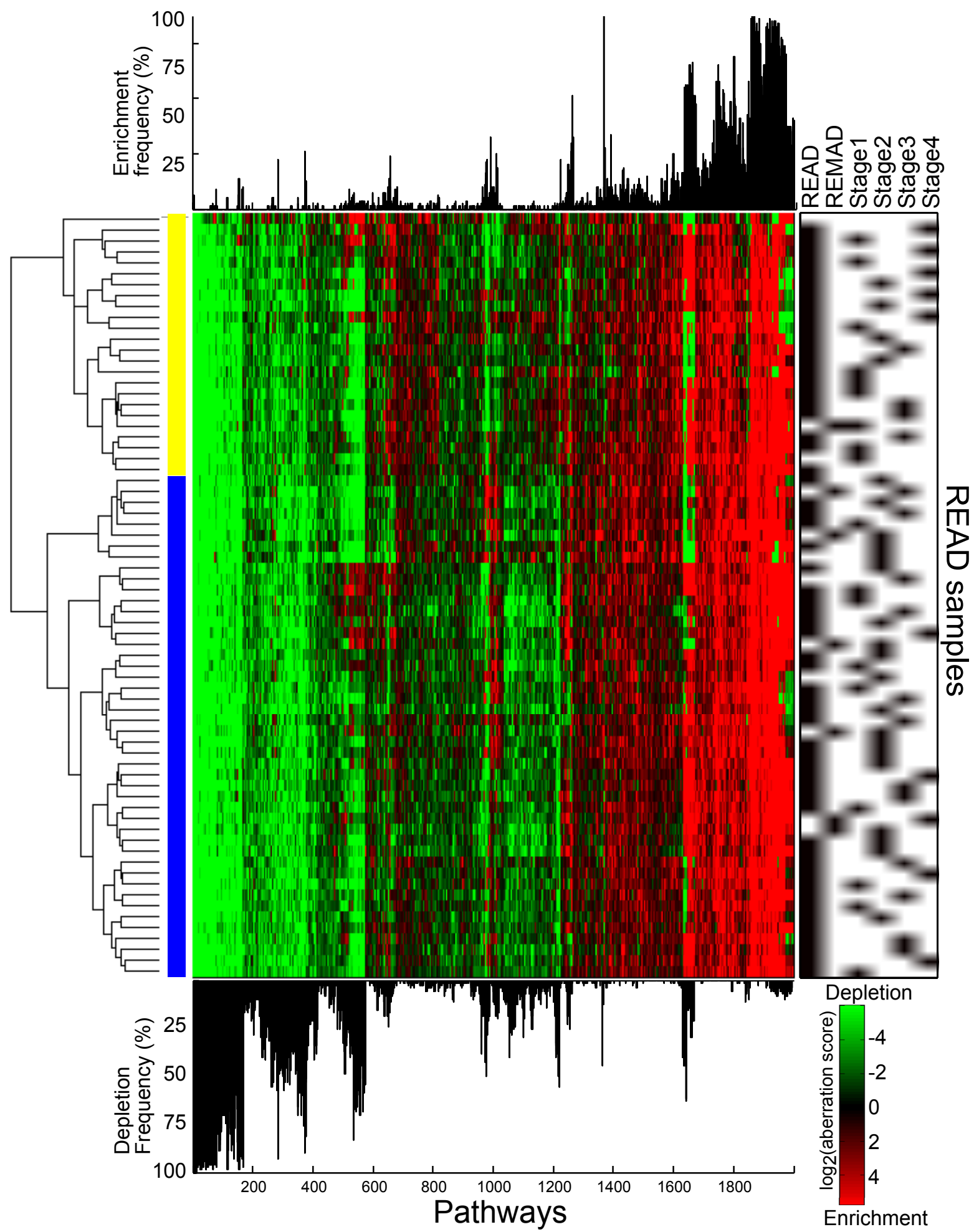

Supplement: Additional file 4 — Supplementary figures [file 1752-0509-7-S1-S1-S4.zip › figS4.pdf]

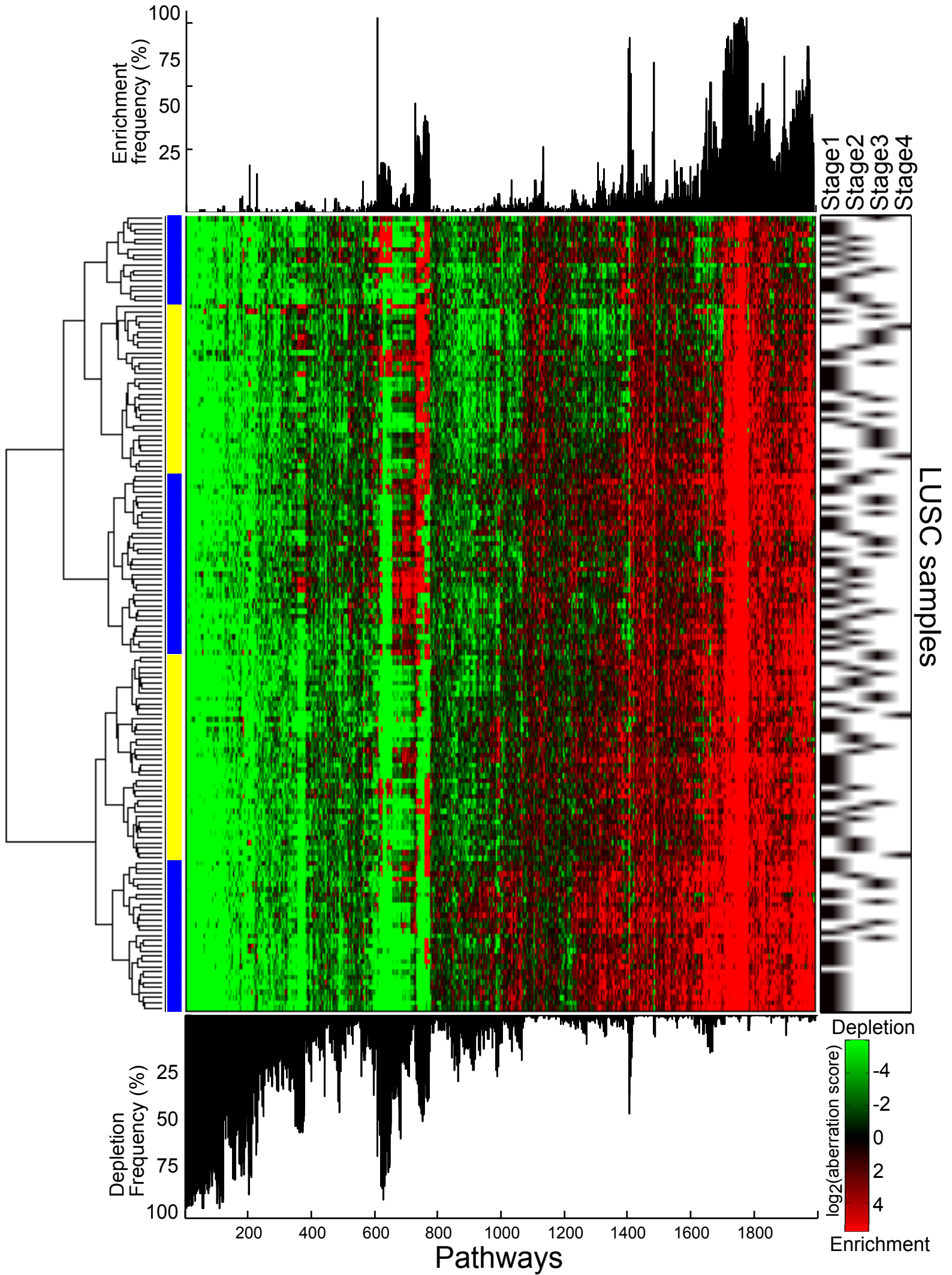

Supplement: Additional file 4 — Supplementary figures [file 1752-0509-7-S1-S1-S4.zip › figS5.pdf]

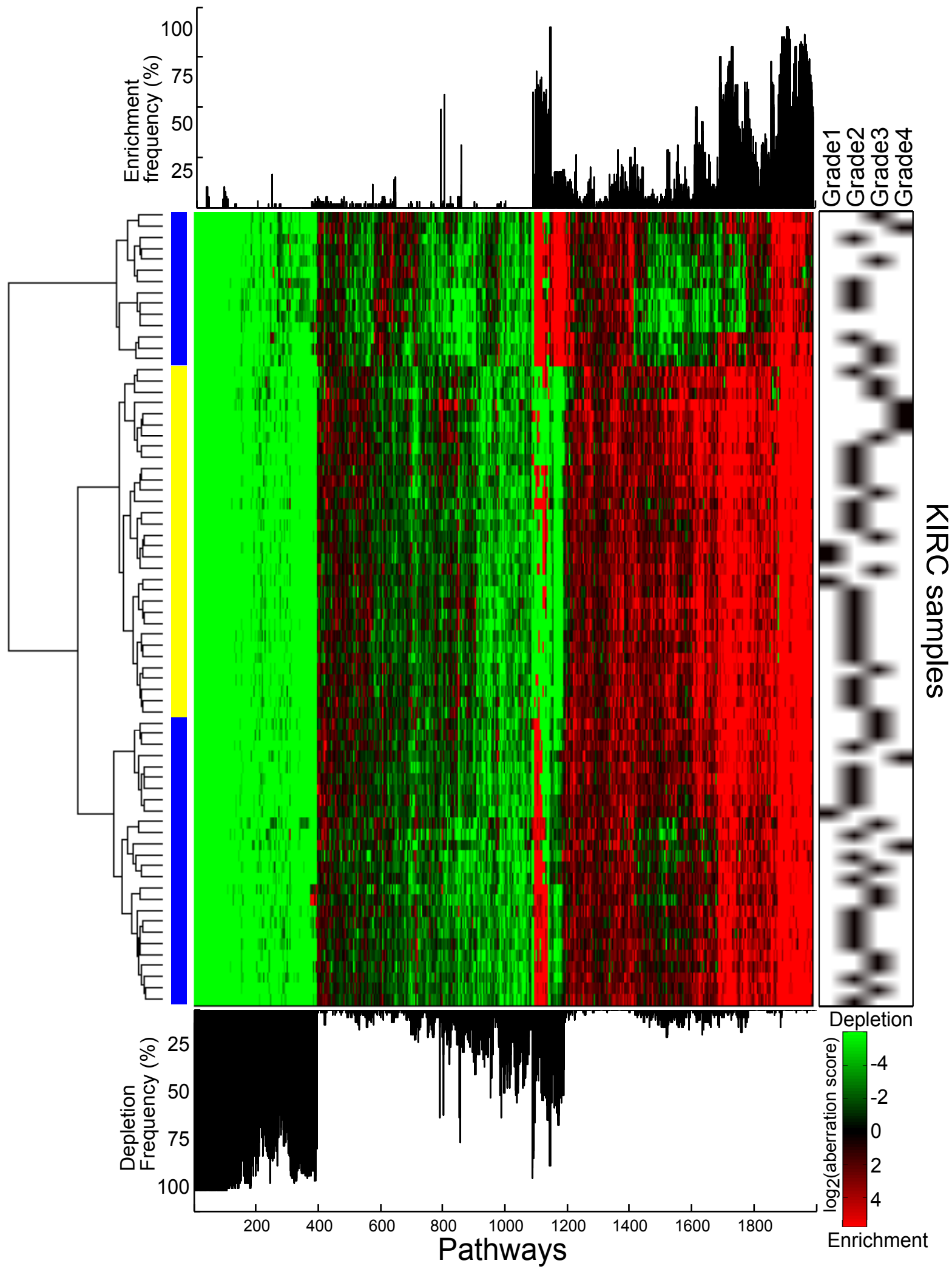

Supplement: Additional file 4 — Supplementary figures [file 1752-0509-7-S1-S1-S4.zip › figS6.pdf]

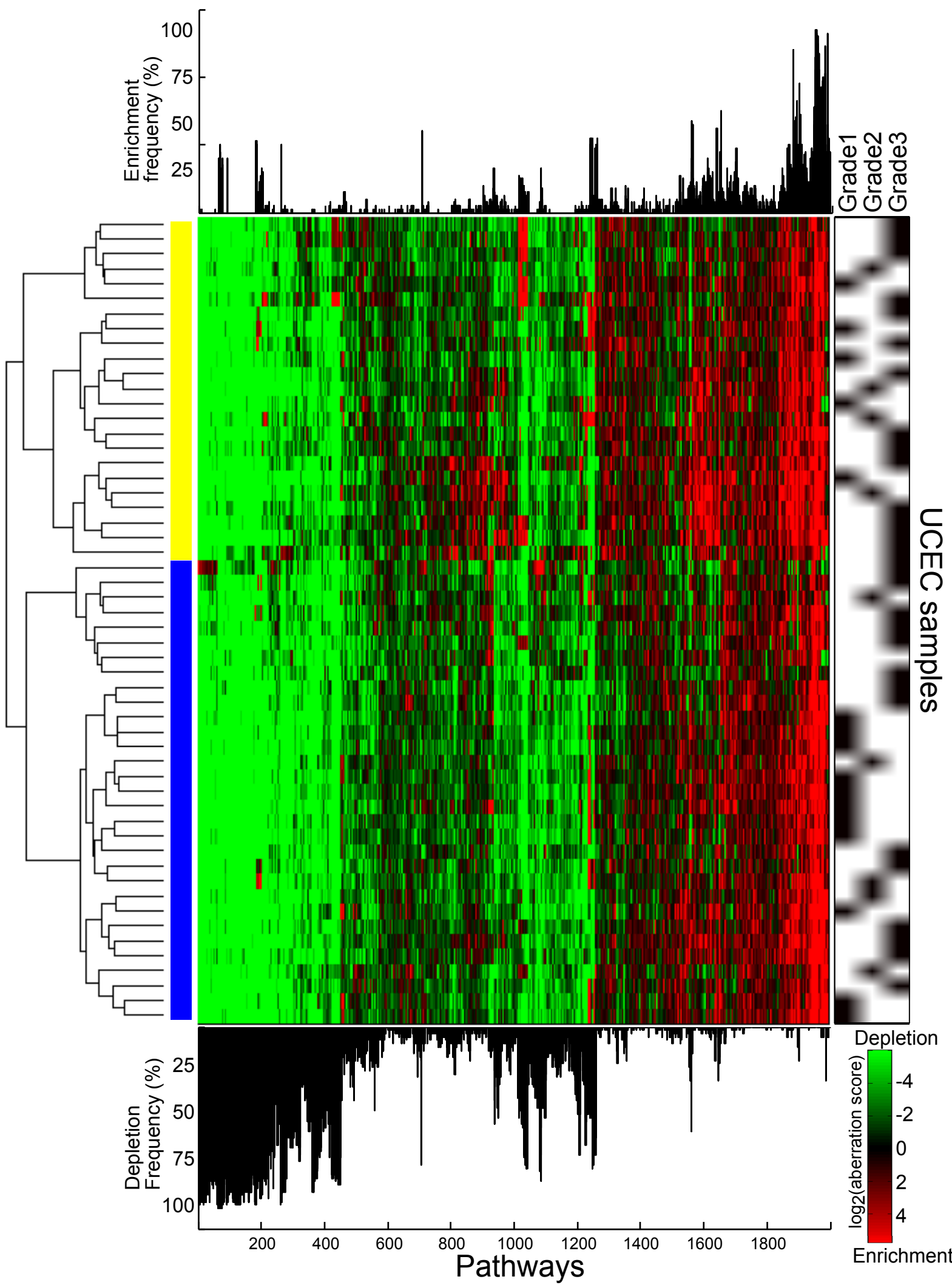

Supplement: Additional file 4 — Supplementary figures [file 1752-0509-7-S1-S1-S4.zip › figS7.pdf]

a)

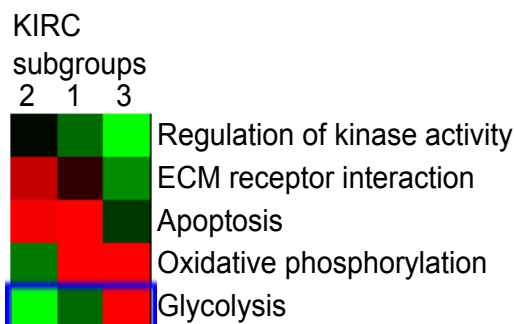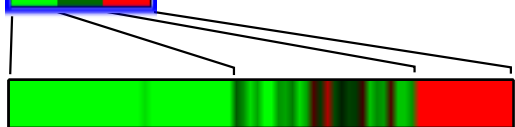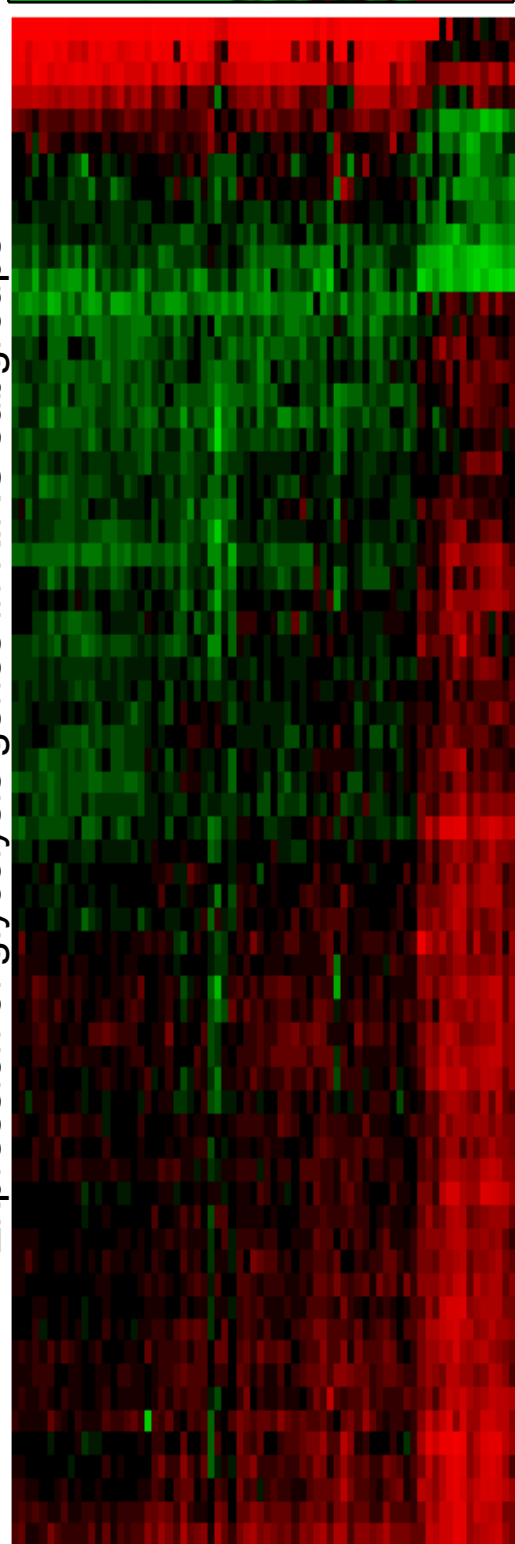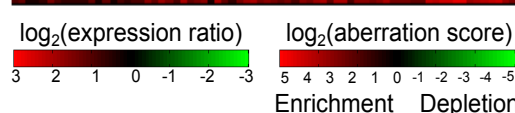

b)

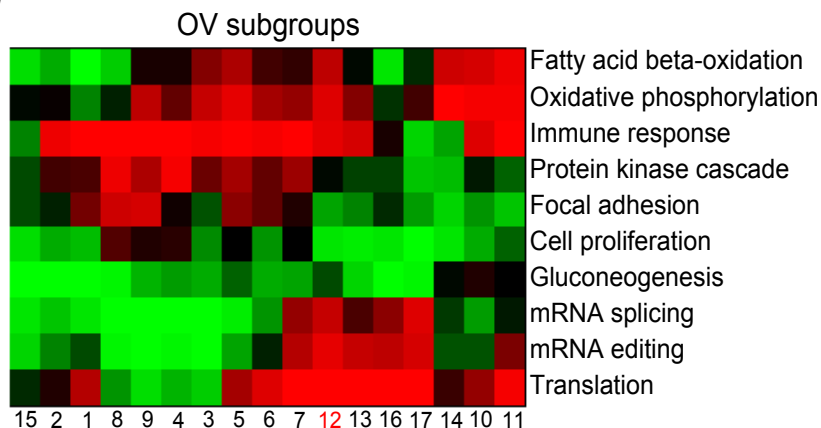

c)

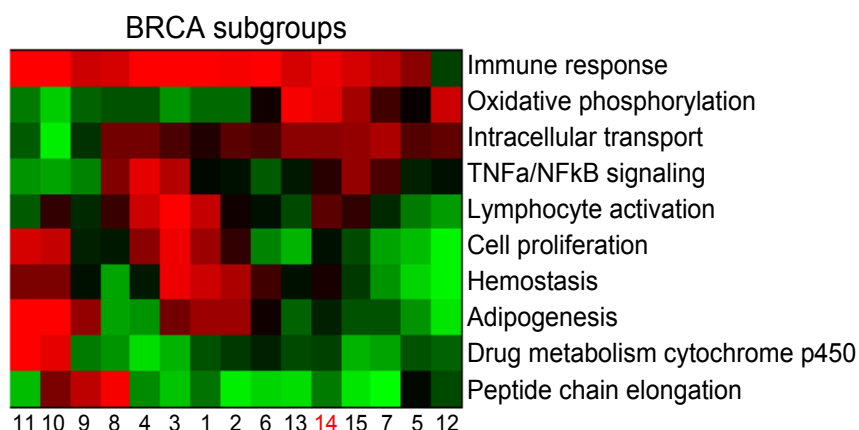

d)

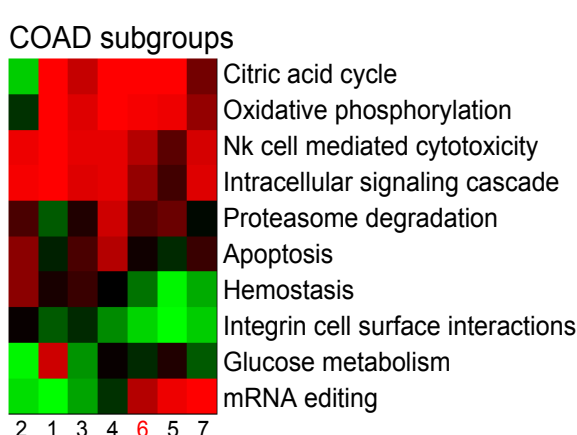

e)

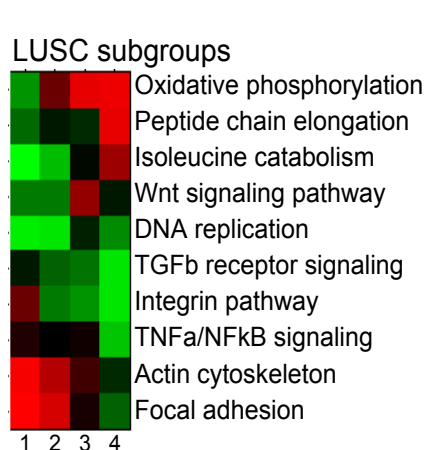

f)

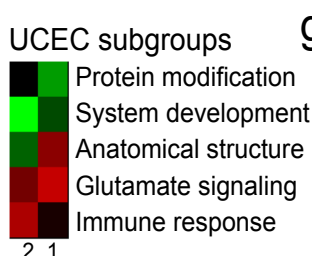

g)

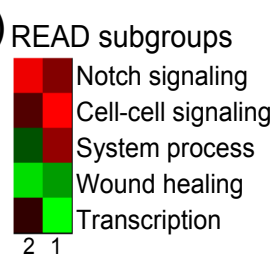

Supplement: Additional file 4 — Supplementary figures [file 1752-0509-7-S1-S1-S4.zip › figS8.pdf]
