# Supplementary material for: Characterization of aberrant pathways across human cancers
Source: BMC Syst Biol. 2013 Aug 12;7(Suppl 1):S1. doi: 10.1186/1752-0509-7-S1-S1 (PMC3750561; doi:10.1186/1752-0509-7-S1-S1)
Supplement: Additional file 4 — Supplementary figures [file 1752-0509-7-S1-S1-S4.zip › Supplemental Figure Legends.doc]

**Supplemental Figure Legends:**

**S1: Clustering of breast cancer pathway aberration profile**

Pathway aberration matrix of BRCA shows enriched (in red) and depleted pathways (in green). The bar plot on top of the matrix shows the frequency of enrichment for each pathway, and the bar plot below shows the frequencies of depletion. Hierarchical clustering of the matrix resulted in 15 subgroups (marked in alternating blue and yellow bars, first subgroup being marked with the first blue bar in the bottom), one of which was more malignant (subgroup 14 in red). Some of the subgroups corresponded to the pre-existing molecular classifications in right.

**S2: Clustering of colon cancer pathway aberration profile**

Pathway aberration matrix of COAD shows enriched (in red) and depleted pathways (in green). The bar plot on top of the matrix shows the frequency of enrichment for each pathway, and the bar plot below shows the frequencies of depletion. Hierarchical clustering of the matrix resulted in 7 subgroups (marked in alternating blue and yellow bars, first subgroup being marked with the first blue bar in the bottom), one of which was more malignant (subgroup 6 in red). Subgroups do not correspond to mucinous subtype (COMAD) or different tumour stages (right).

**S3: Clustering of ovarian cancer pathway aberration profile**

Pathway aberration matrix of OV shows enriched (in red) and depleted pathways (in green). The bar plot on top of the matrix shows the frequency of enrichment for each pathway, and the bar plot below shows the frequencies of depletion. Hierarchical clustering of the matrix resulted in 17 subgroups (marked in alternating blue and yellow bars, first subgroup being marked with the first blue bar in the bottom), one of which was more malignant (subgroup 12 in red). Subgroups do not correspond to different tumour stages (right).

**S4: Clustering of rectal cancer pathway aberration profile**

Pathway aberration matrix of OV shows enriched (in red) and depleted pathways (in green). The bar plot on top of the matrix shows the frequency of enrichment for each pathway, and the bar plot below shows the frequencies of depletion. Hierarchical clustering of the matrix resulted in 2 subgroups (marked in alternating blue and yellow bars, first subgroup being marked with the first blue bar in the bottom). Subgroups do not correspond to mucinous subtype (REMAD) or different tumour stages (right).

**S5: Clustering of lung cancer pathway aberration profile**

Pathway aberration matrix of OV shows enriched (in red) and depleted pathways (in green). The bar plot on top of the matrix shows the frequency of enrichment for each pathway, and the bar plot below shows the frequencies of depletion. Hierarchical clustering of the matrix resulted in 5 subgroups (marked in alternating blue and yellow bars, first subgroup being marked with the first blue bar in the bottom). Subgroups do not correspond to different tumour stages (right).

**S6: Clustering of kidney cancer pathway aberration profile**

Pathway aberration matrix of OV shows enriched (in red) and depleted pathways (in green). The bar plot on top of the matrix shows the frequency of enrichment for each pathway, and the bar plot below shows the frequencies of depletion. Hierarchical clustering of the matrix resulted in 3 subgroups (marked in alternating blue and yellow bars, first subgroup being marked with the first blue bar in the bottom). Subgroups do not correspond to different tumour grades (right).

**S7: Clustering of endometrial cancer pathway aberration profile**

Pathway aberration matrix of OV shows enriched (in red) and depleted pathways (in green). The bar plot on top of the matrix shows the frequency of enrichment for each pathway, and the bar plot below shows the frequencies of depletion. Hierarchical clustering of the matrix resulted in 2 subgroups (marked in alternating blue and yellow bars, first subgroup being marked with the first blue bar in the bottom). Subgroups do not correspond to different tumour grades (right).

**S8: Differential pathway and gene expression patterns in the cancers**

a) There are significant differences in aberration frequency of several pathways in three KIRC subgroups, including the five pathways shown here on top. As an example, highlighted in blue is the glycolysis pathway that is very frequently depleted in subgroup 2, moderately depleted in subgroup 1, and enriched in subgroup 3. Aberration statuses of individual samples in each subgroup are shown on top of the heatmap of gene expression levels of glycolysis pathway genes. A very distinct gene expression signature is observed for subgroup 3. DPYS, UPB1 and PPAP2B are consistently upregulated in samples where glycolysis pathway is not enriched, whereas a group of eight downregulated genes (ACLY, PFKFB3, MLXIPL, SLC35D1, TPI1, RPIA, AMPD2, and PFKB4) are a characteristic of the glycolysis-enriched subgroup 3 in addition to a fair amount of upregulated genes, including subunits of NADH dehydrogenase complex, ATP synthase complex, cytochrome c oxidase, and ubiquinol-cytochrome reductase. The most prominently different pathway aberration rates are shown for subgroups of b) OV, c) BRCA, d) COAD, e) LUSC, f) UCEC, and g) READ.
